# Web Portals for Patients With Chronic Diseases: Scoping Review of the Functional Features and Theoretical Frameworks of Telerehabilitation Platforms

**DOI:** 10.2196/27759

**Published:** 2022-01-27

**Authors:** Yuh Morimoto, Tetsuya Takahashi, Ryuichi Sawa, Masakazu Saitoh, Tomoyuki Morisawa, Nobuyuki Kagiyama, Takatoshi Kasai, Birthe Dinesen, Malene Hollingdal, Jens Refsgaard, Hiroyuki Daida

**Affiliations:** 1 Faculty of Health Science Juntendo University Tokyo Japan; 2 Department of Physical Therapy Faculty of Health Science Juntendo University Tokyo Japan; 3 Department of Digital Health and Telemedicine Research and Development Faculty of Health Science Juntendo University Tokyo Japan; 4 Department of Cardiovascular Biology and Medicine Juntendo University Graduate School of Medicine Tokyo Japan; 5 Laboratory for Welfare Technologies - Telehealth & Telerehabilitation Sport Sciences - Performance and Technology, Department of Health Science and Technology Aalborg University Aalborg Denmark; 6 Department of Cardiology Regional Hospital in Viborg Viborg Denmark

**Keywords:** telerehabilitation, web portal, chronic disease, monitoring/data tracking function, patient-centered care

## Abstract

**Background:**

The COVID-19 pandemic has required an increased need for rehabilitation activities applicable to patients with chronic diseases. Telerehabilitation has several advantages, including reducing clinic visits by patients vulnerable to infectious diseases. Digital platforms are often used to assist rehabilitation services for patients in remote settings. Although web portals for medical use have existed for years, the technology in telerehabilitation remains a novel method.

**Objective:**

This scoping review investigated the functional features and theoretical approaches of web portals developed for telerehabilitation in patients with chronic diseases.

**Methods:**

PubMed and Web of Science were reviewed to identify articles associated with telerehabilitation. Of the 477 nonduplicate articles reviewed, 35 involving 14 portals were retrieved for the scoping review. The functional features, targeted diseases, and theoretical approaches of these portals were studied.

**Results:**

The 14 portals targeted patients with chronic obstructive pulmonary disease, cardiovascular, osteoarthritis, multiple sclerosis, cystic fibrosis diseases, and stroke and breast cancer survivors. Monitoring/data tracking and communication functions were the most common, followed by exercise instructions and diary/self-report features. Several theoretical approaches, behavior change techniques, and motivational techniques were found to be utilized.

**Conclusions:**

The web portals could unify and display multiple types of data and effectively provide various types of information. Asynchronous correspondence was more favorable than synchronous, real-time interactions. Data acquisition often required assistance from other digital tools. Various functions with patient-centered principles, behavior change strategies, and motivational techniques were observed for better support shifting to a healthier lifestyle. These findings suggested that web portals for telerehabilitation not only provided entrance into rehabilitation programs but also reinforced participant-centered treatment, adherence to rehabilitation, and lifestyle changes over time.

## Introduction

Chronic diseases are the leading causes of death worldwide. The World Health Organization has reported that chronic diseases are responsible for almost 60% of deaths worldwide [1]. Studies show evidence of the effectiveness of rehabilitation for patients with chronic disease; however, rehabilitation programs are generally underused [2,3]. The major barriers to participation in a rehabilitation program include inconvenient timing, travel and transport issues, and lack of perceived benefit [4]. Currently, rehabilitation services are facing even more difficulty because of the COVID-19 pandemic. Conventional clinic-based rehabilitation programs have been suspended as a result of physical distancing recommendations and a shortage of health care services [5,6]. Patients with chronic diseases are more likely to develop severe conditions than patients without chronic diseases, thus visiting clinics is considered to be a risk to be avoided by patients [7]. Telerehabilitation can address such obstacles, even during the COVID-19 pandemic.

Telerehabilitation is defined as rehabilitation activities performed using information provided by communication technologies over a distance [8]. Telerehabilitation for patients with chronic diseases may consist of many components, for example, exercise instruction, education, better communication, and self-management training [9-11]. Digital platforms are often used to assist rehabilitation services for patients in remote settings [12-14], and web portals are one of the potential technologies. The term “portal (computing)” has been described as a website that is used as a gateway to the internet, where information useful to a person interested in particular topics has been gathered [15]. A web portal in clinical use has been described as a kind of electronic health record that permits patients to access their records or communicate with their health care professionals [16,17]. Furthermore, the US government describes a web portal as “a secure online website that gives patients convenient, 24-hour access to personal health information from anywhere with an internet connection” [18]. Taking into account the personal nature of the information, access is often limited to authorized people in a secure and confidential setting [19]. To encourage patient engagement and provide benefits, web portals are recommended to follow the principle of patient-centered care [20]. The primary concept of patient-centered care is that patient values guide clinical decisions [21]. The 6 factors defined as components of patient-centered care are (1) respect for patients’ values, preferences, and expressed needs; (2) coordination and integration of care; (3) information, communication, and education; (4) physical comfort; (5) emotional support; and (6) involvement of family and friends [22].

Web portals have the potential to be a core digital component for patient-centered telerehabilitation, however, relatively few have been adapted for telerehabilitation [23], and few studies to date have analyzed the use of web portals for telerehabilitation in an international context. It is not known whether there are unique characteristics in web portals developed for telerehabilitation targeting chronic diseases rather portals designed for other medical purposes. For these reasons, a scoping review was conducted to gather knowledge about what has been designed and tested in clinical practice, as well as to identify any existing gaps in knowledge. The search strategy according to population, concept, and context elements was as follows: P, telerehabilitation participants with chronic disease; C, web portals developed for telerehabilitation, functional features, theoretical approaches, behavior change techniques (BCT), motivational techniques (MTs), and mode of delivery; C, any gender, age, or region. The following research question was formulated to guide this review: What are the functional features and theoretical approaches of web portals developed for telerehabilitation programs in patients with chronic diseases, as well as any characteristics that can be observed through the investigations?

## Methods

### Study Design

This study was designed to map the functional features of web portals utilized for telerehabilitation targeting chronic diseases. Although systematic reviews are preferred for answering clearly defined questions, a scoping review method is considered useful for answering broad questions [24]. The scoping review protocol in this study was developed using the guidelines provided by the Preferred Reporting Items for Systematic Reviews and Meta-analysis Extension for Scoping Reviews (PRISMA-ScR) checklist [24,25]. With the cooperation of an experienced university librarian, multiple databases were searched, ensuring the appropriateness of the search strategy. The final version of the protocol is available from a protocol registry [26].

### Identification and Selection of the Relevant Articles

A literature search of the international online bibliographic databases PubMed [27] and the Web of Science [28] was performed on May 17, 2021. The search formula used for the PubMed search engine was ((telerehabilitation) AND (chronic)) OR ((telerehabilitation) AND ((portal) OR (web-based) OR (digital platform) OR (online platform) OR (internet-based))). Because the definitions of web portals for telerehabilitation participants were not determined in a common context, digital platforms used for telerehabilitation were reviewed with the principal concepts of web portals described above, such as (1) web-based application, (2) allows the participants 24-hour access, and (3) containing useful rehabilitation-related functions for participants. To extract core components of web portals for telerehabilitation, portals developed for purposes other than telerehabilitation were excluded, as were clinician portals. To focus on the essential features, telerehabilitation portals targeting anything other than chronic diseases were also excluded. Web applications designed for a single purpose, web services with limited time of access, and digital platforms based on native applications (eg, iOS and Android apps and PC applications) were excluded based on the definitions of web portals described above. Abstracts of the selected studies were reviewed by the team members according to the inclusion/exclusion criteria. Review articles, editorials, conference reports, and interviews were also excluded, whereas protocols were included.

Data extraction and charting were performed according to Arksey and O’Malley’s guideline [29]. Data from the selected articles were extracted with the following modules: portal name, project name, country, functional features, targeted chronic diseases, language, and other systems required for the telerehabilitation service. Theories and models used in a study were investigated as well as BCTs, MTs, and mode of delivery. Several articles were found to describe the same telerehabilitation project. The names of the projects and web portals, as well as the authors, institutions, and trial registration numbers, were used to identify telerehabilitation projects described in multiple articles.

## Results

### Overview

**Figure 1:**
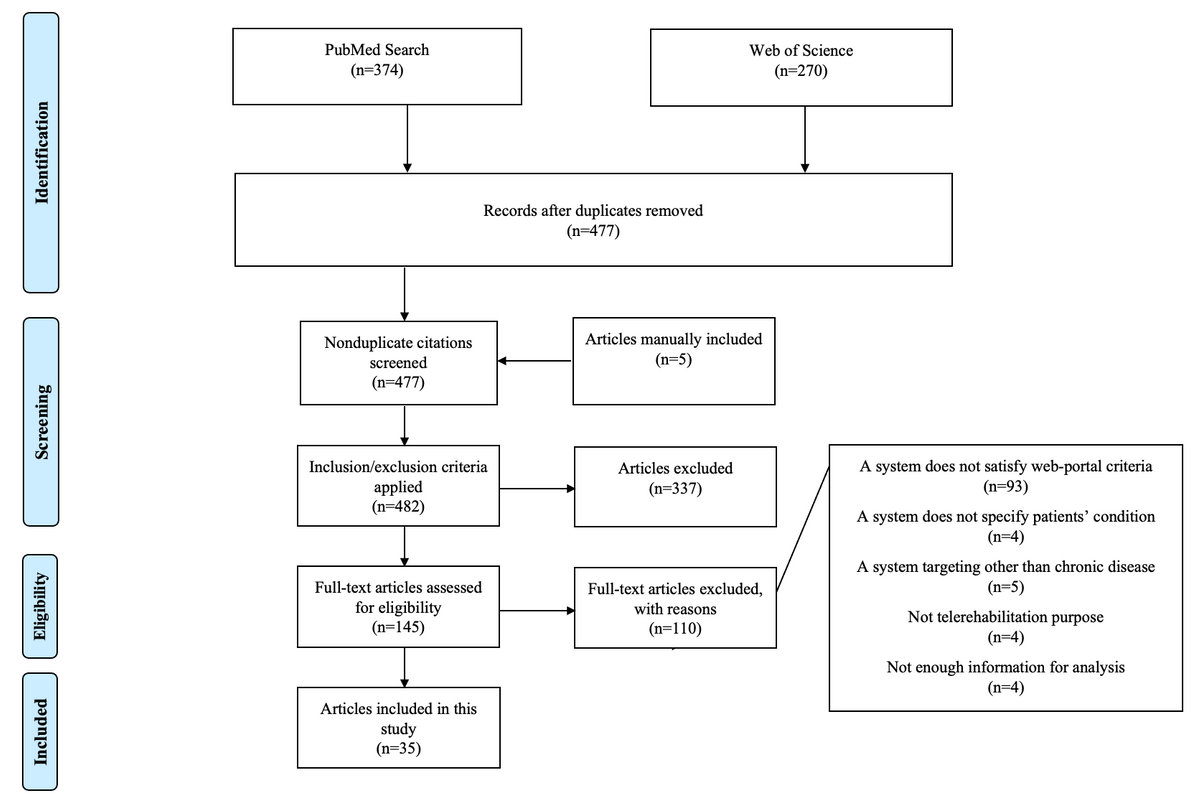
Flow diagram of article identification.

Of the 644 articles initially identified by keywords, 477 remained after the removal of duplicates. An additional 5 articles were found through manual searching. Figure 1 shows the flow diagram of the article identification process. The titles and abstracts of these 482 nonduplicated articles were reviewed during the initial screening phase, resulting in the identification of 145 articles for full-text screening. Of these 145 articles, 35 articles with 14 platforms satisfied the criteria and were included in the analysis. Full reasons for exclusion are shown in Figure 1.

### Functional Features of Telerehabilitation Portals

**Figure 2:**
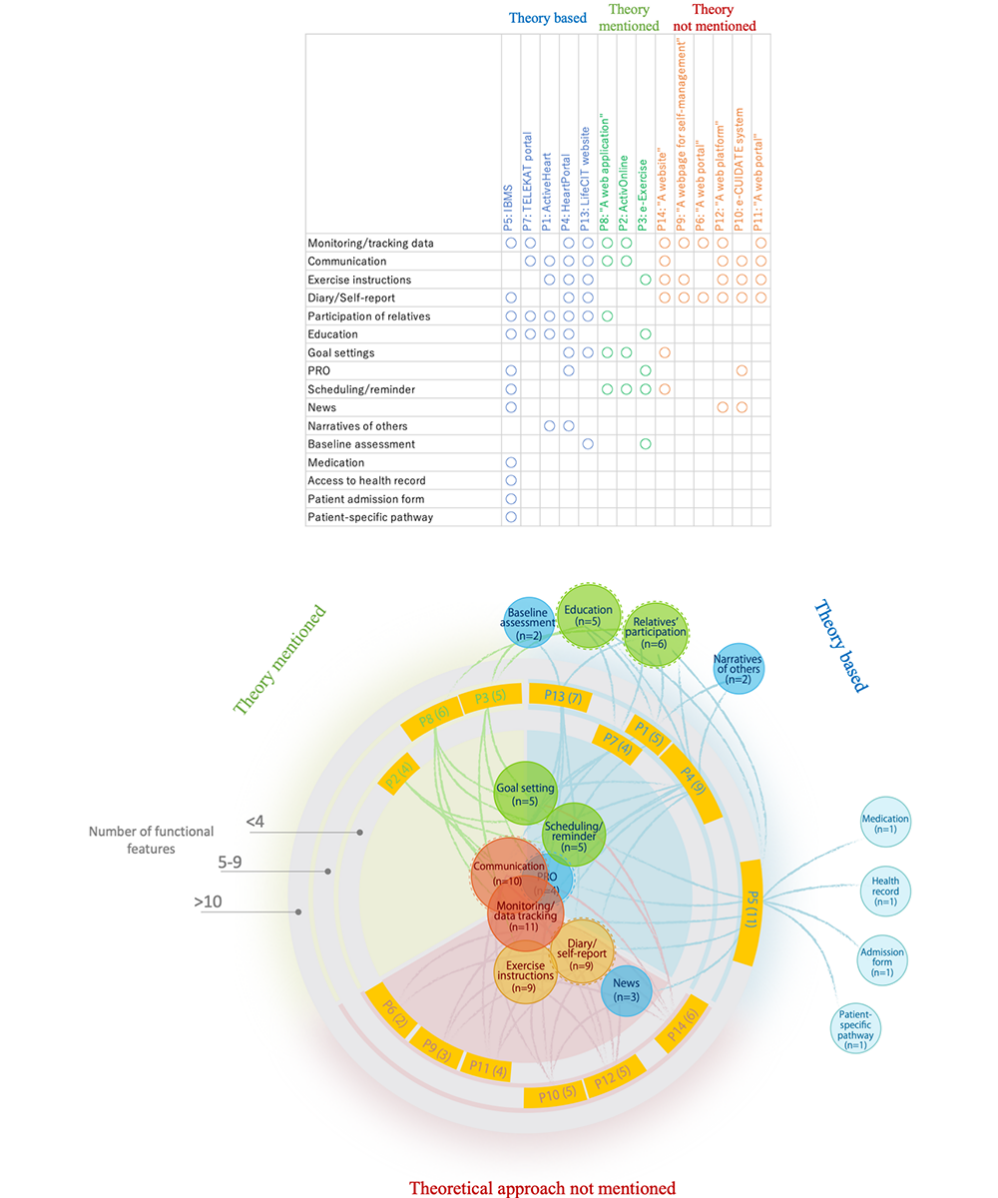
A summary of functional features and the 14 portals. A colored circle indicates a functional feature. The number in a circle denotes the number of portals that employ the functional feature. A colored circle with a dotted line is associated with the principle of patient-centered care. Yellow boxes on a gray circle indicate a web portal. The number in parenthesis denotes the number of functional features the portal implemented. P1: ActiveHeart; P2: ActivOnline; P3: e-Exercise; P4: HeartPortal; P5: IBMS; P6: “A web portal”; P7: TELEKAT portal; P8: “A web application”, P9: “A webpage for self-management”; P10: e-CUIDATE system; P11: “A web portal”; P12: “A web platform; P13: LifeCIT website; P14: “A website”. IBMS: Integrated Care Portal Multiple Sclerosis; TELEKAT: telehomecare, chronic patients, and the integrated health care system; P: portal; PROs: patient reported outcomes.

Five portals were for patients with chronic obstructive pulmonary disease (COPD) [30-43], 3 were for patients with cardiovascular diseases [44-49], 2 for multiple sclerosis [50,51], and 1 each for patients with osteoarthritis [52-56], cystic fibrosis [57-59], and stroke [60], and breast cancer survivors [61-64]. These 14 portals were implemented with multiple functions. Monitoring/data tracking functions and communication platforms were the most common features, provided by 11 and 10 portals, respectively. Monitoring/tracking functions foster self-management skills in patients by gaining knowledge and awareness of their health and active involvement in rehabilitation programs [30,37,46]. Communication functions take various forms, including text messaging systems, web forums, and videoconferencing. Exercise instruction function with audiovisual contents was emphasized in 9 portals, and diary/self-report function was found in 9 portals, being used for patients to share their rehabilitation reports and experience with health care team members. Six portals allowed relatives to participate in the rehabilitation program with permission of the patient. Education was also frequently found with 5 portals. Patient-reported outcomes (PROs) data and goal settings can be collected or determined through specifically designed systems [55], as well as through diary or communication functions [48,50,64]. Two portals embedded audiovisual contents about other patients and their family members talking about their experiences of rehabilitation (narrative of others) [46,48]. Although monitoring is one of the major functions, portals often require assistance from other digital tools [30,35,41,44,50] or extra effort of manual operations [49,57]. The most dominant security measure was an individual, password-protected system. Furthermore, ActivOnline protects user data and privacy by employing security measures (eg, 128-bit SSL security) to provide encrypted data transfer [59], and “web application” (portal 8) employed a 2-factor log-in system with participants’ private mobile phone [49]. The summary of functional features and the 14 portals are shown in Table 1 and Figure 2. Detailed descriptions of each portal are given in Multimedia Appendix 1.

Some functional features are considered to be associated with the principle of patient-centered care. Respect for patients’ values can be attempted by PRO, diary function, and the participatory design process, which several portals followed [36,45,47,50]. Communication and education functions can provide coordination and integration of care, and the function of the relatives’ participation is associated with involvement of family and friends.

**Table 1:** A list of included studies.

Portal name and references		Project name	Diseases	Functional features	Required additional systems
**Portal 1: ActiveHeart**		TTP^a^	Heart disease	Communication, exercise instructions, education, participation of relatives, narratives of others^b^	The shared care platform, MyMedic, Fitbit, sphygmomanometer, scale
	Dinesen et al [44]				
	Dinesen et al [45]				
	Melholt et al [46]				
**Portal 2: ActivOnline**		ActionPACT^c^	Cystic fibrosis (later used for COPD^d^ and bronchiectasis)	Monitoring/tracking data, communication, goal settings, scheduling/reminder	Step counter (Fitbit, mobile phone or pedometer)
	Cox et al [57]				
	Liacos et al [58]				
	Cox et al [59]				
**Portal 3: e-Exercise**		e-Exercise	Knee/hip osteoarthritis	Exercise instructions, education, PROs^e^, scheduling/reminder, baseline assessment	Face-to-face sessions
	Kloek et al [52]				
	Kloek et al [53]				
	Kloek et al [54]				
	de Vries et al [55]				
	Bossen et al [56]				
**Portal 4: HeartPortal**		Future patient	Heart failure	Monitoring/tracking data, communication, exercise instructions, diary, education, participation of relatives, goal settings (via communication and diary function), PROs, narratives of others	Sphygmomanometer, scale, data transmitter, step counters, sleep sensor, iPad
	Joensson et al [47]				
	Dinesen et al [48]				
**Portal 5: IBMS^f^**		—^g^	Multiple sclerosis	Monitoring/tracking data, diary, education, participation of relatives, PROs (via diary function), scheduling/reminder, news, medication, access to a health care record (multiple sclerosis care record), patient administration form, patient-specific pathways	MSDS^3Dh^, multiple sclerosis case record
	Voigt et al [50]				
**Portal 6: “A web portal”**		—	COPD	Monitoring/tracking data, diary	Activity coach (smartphone and accelerometer)
	Tabak et al [30]				
**Portal 7: TELEKAT^i^ portal**		TELEKAT	COPD	Monitoring/tracking data, communication, education (via communication function), participation of relatives	MyMedic/MyMedicPlus, scale, sphygmomanometer, oximeter, spirometer
	Dinesen et al [31]				
	Haesum et al [32]				
	Dinesen et al [33]				
	Jensen et al [34]				
	Dinesen et al [35]				
	Dinesen et al [36]				
	Huniche et al [37]				
**Portal 8: “A web application”**		SmartCareCAD	Coronary artery disease	Monitoring/tracking data, communication, participation of relatives, goal setting, scheduling/reminder	Mio alpha, hip-worn accelerometer
	Brouwers et al [49]				
**Portal 9: “A webpage for self-management”**		—	COPD	Monitoring/tracking data, exercise instructions, diary	Treadmill, iPad, iPad holder, web conference application, pulse oximeter
	Zanaboni et al [38]				
	Hoaas et al [39]				
	Zanaboni et al [40]				
**Portal 10: e-CUIDATE system**		e-CUIDATE	Breast cancer survivors	Communication, exercise instructions, diary, PROs (via communication function), news	Telephone call
	Ariza-Garcia et al [61]				
	Galiano-Castillo et al [62]				
	Galiano-Castillo et al [63]				
	Galiano-Castillo et al [64]				
**Portal 11: “A web portal”**		—	COPD	Monitoring/tracking data, communication, exercise instructions, diary	Activity coach (smartphone and accelerometer)
	Tabak et al [41]				
**Portal 12”: “A web platform”**		—	Multiple sclerosis	Monitoring/tracking data, communication, exercise instructions, diary, news	An ad hoc tracking system for determination of posture, ToF camera^j^
	Eguiluz-Perez and Garcia-Zapirain [51]				
**Portal 13: LifeCIT^k^ website**		LifeCIT	Poststroke	Monitoring/tracking data, communication, exercise instructions, diary, participation of relatives, goal setting, baseline assessment	C-Mitt (a glove to restrict functional hand movement)
	Burridge et al [60]				
**Portal 14: “A website”**		iTrain	COPD	Monitoring/tracking data, communication, exercise instructions, diary, goal setting, scheduling/reminder	Treadmill, iPad, iPad holder, web conference application, pulse oximeter
	Hoaas et al [42]				
	Zanaboni et al [43]				

^a^TTP: teledialog telerehabilitation program.

^b^Interviews, stories, and experiences of other patients or relatives who have the same disease.

^c^ActionPACT: the active online physical activity in the cystic fibrosis trial.

^d^COPD: chronic obstructive pulmonary disease.

^e^PROs: patient-reported outcomes.

^f^IBMS: integrated care portal multiple sclerosis.

^g^Not specified.

^h^Integration of the multiple sclerosis documentation system.

^i^TELEKAT: telehomecare, chronic patients, and the integrated health care system.

^j^Time-of-flight camera.

^k^Constraint-induced therapy.

### Theoretical Approaches of Telerehabilitation Portals

Theoretical approaches were observed in 8 portals. Among them, *eHealth literacy* described by Norman and Skinner [65], Wenger’s *Communities of Practice* [66], and *Self-Determination Theory* [67] were used for both development process and qualitative usability evaluation [31,44,47,48]. The model of *eHealth literacy* was first used in the qualitative data analysis in ActiveHeart [46], and then employed in HeartPortal to foster participants’ empowerment [48]. *Communities of practice* focus on people who share a common interest or concern and interact regularly, with learning taking place during interpersonal interactions [66]. The theory influenced the design of portals as facilitating communication and knowledge-sharing functions among participants and patients with chronic diseases in the ActiveHeart and telehomecare, and the integrated health care system (TELEKAT) portals [31,44]. The Self-Determination Theory takes into account intrinsic and extrinsic motivation with 3 basic human needs: autonomy, competence, and relatedness [67]. HeartPortal includes a graphical function that displays charts of each participant’s physical data [48]. Participants who monitor their condition using detailed charts are expected to gain autonomy and competence. Relatives’ participation upon patient’s permission is included in several portals, and it is in the context of relatedness in HeartPortal. *Pathway-based care model*–implemented integrated care portal multiple sclerosis (IBMS) is an approach that provides a patient with a complete picture of his/her disease progression and the current state of an evidence-based treatment strategy [50,68]. By using this model, milestones of the treatment are defined, and shared decision making between patients and the multidisciplinary care team is expected to be induced [50]. *Person-based approach* for designing successful digital interventions [69] was the method used to guide the development phase of LifeCIT’s website [60].

Some studies were not “theory based” but introduced theories in their study concept, thus we considered them as “theory mentioned.” *Cognitive behavior principles* were mentioned in 3 studies [49,53,57], where 1 study [49] briefly mentions theory linking to relapse prevention. *Operant conditioning* was associated with time-contingent exercise activity in e-Exercise [56]. On the contrary, “A webpage for self-management” (portal 9) stated their study was empirically based. Moreover, e-Exercise, “A web portal” (portal 11), and “A website” (portal 14) used empirical data of their previous trials [41,56].

The BCTs and MTs were identified by the authors’ descriptions in the included articles, as well as referring to a taxonomy of BCTs proposed by Abraham and Michie [70]. The incorporation of several BCTs was reported to be more effective than interventions that incorporated fewer techniques [71]. Relatives’ participation and goal settings were commonly found in portals that took theoretical approaches. The general purpose of BCTs is to better support patients shifting to a healthier lifestyle. Note that a technique mentioned as BCT in one study can be implemented in another study without being stated as a BCT purpose.

**Figure 3:**
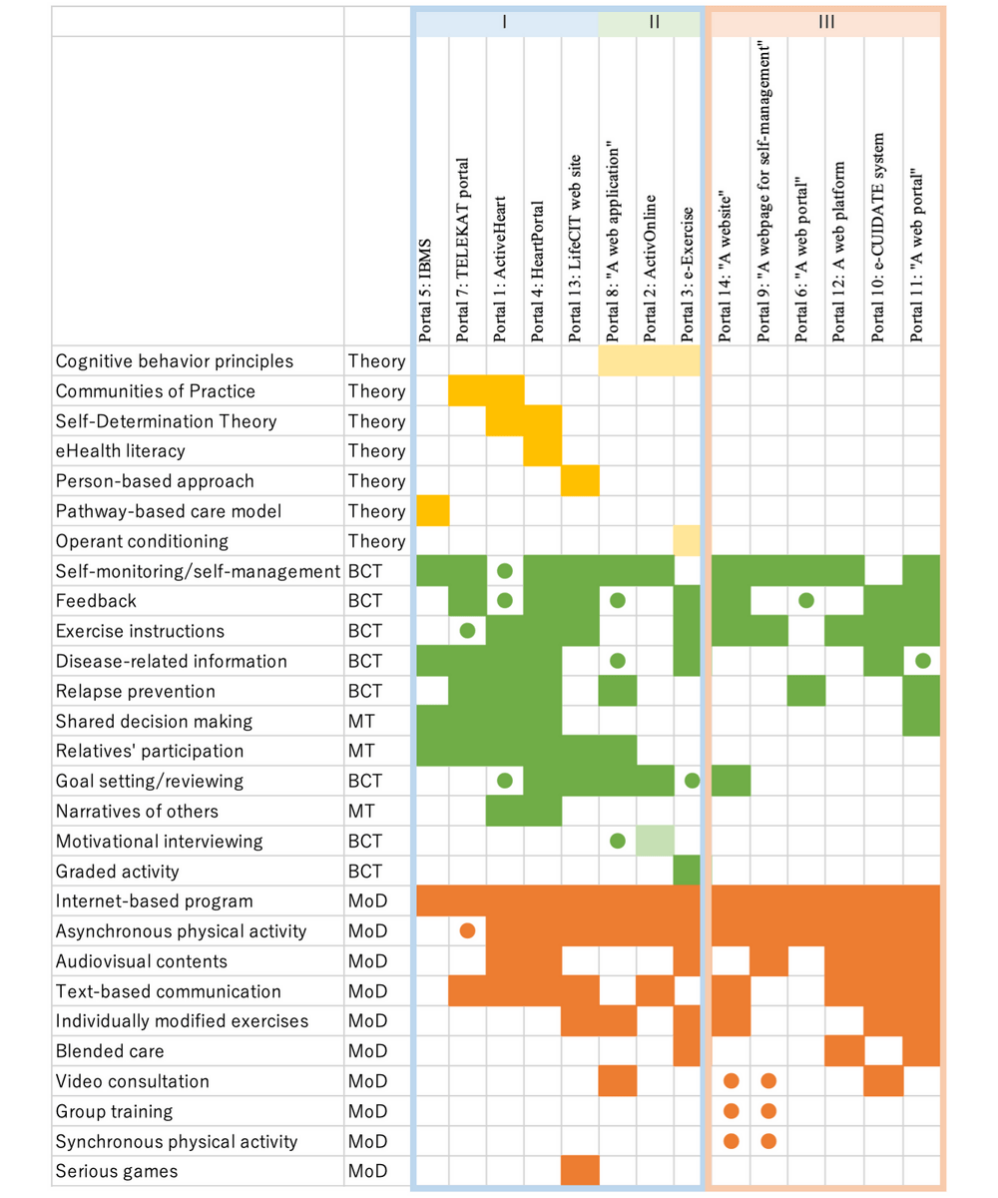
Theoretical approaches, BCTs, MTs, and modes of delivery employed in the 14 portals. Yellow square, theory applied; light yellow squares, theory or model mentioned without specifies on the mode of implication; green squares, BCT applied; light green squares, BCT mentioned without specifies on the mode of implication; green circles, through an additional system; orange squares, mode of delivery applied; orange circles, through an additional system including another digital platform used simultaneously, video consultation with HCP, or face-to-face session as part of the intervention; I: theory based; II: theory mentioned; III: theory not mentioned; BCT: behavior change technique; HCP: health care provider; IBMS: integrated care portal multiple sclerosis; MT: motivational technique; MoD: mode of delivery; TELEKAT: telehomecare, chronic patients, and the integrated health care system.

Mode of delivery is also known to influence behavior [71]. It is obvious that all 14 portals are internet-based programs, of which ActivOnline, “A web application” (portal 8), e-CUIDATE, and “A website” (portal 14) chose this mode of delivery with the expectation of better treatment results compared with conventional center-based treatment [43,49,57,61]. As a synchronous communication, 2 portals embedded a videoconferencing function, which is used for consultations rather than exercise instructions. Asynchronous physical activity was intentionally chosen for better adherence to the exercise in several studies [39,41,43,56,64]. Although 2 interventions applied synchronous group exercises in addition to asynchronous exercises thorough a videoconferencing application apart from a portal [39,43], no functional features of the 14 portals were used for synchronous exercise instructions. The LifeCIT website has gaming contents for poststroke exercise [60]. While the portals that took the theoretical approach tend to have more BCTs, the number of modes of delivery did not differ between portals with or without a theoretical approach. Figure 3 summarizes the theoretical approaches, BCTs, MTs, and modes of delivery employed in the 14 portals.

## Discussion

### Principal Findings

The objective of this scoping review was to investigate functional features and theoretical approaches of web portals developed for telerehabilitation programs in patients with chronic diseases, as well as any characteristics that can be observed through the investigation. As a result, monitoring/tracking data were found to be the most common function in telerehabilitation portals. Data monitoring by patients allowed them to gain self-management skills, which is a key for controlling chronic diseases [11]. Moreover, portal 9 was called by the authors “A webpage for self-management.” Those results indicate that fostering self-management skills is one of the characteristic features of telerehabilitation web portals. Another characteristic feature can be observed through a communication function, because patients with chronic symptoms often require a multidisciplinary care team, including patients’ relatives [48,50]. Facilitating physical activities is another characteristic function of telerehabilitation portals, and exercise programs are often individually modified [41,43,49,56,60,64]. Nine portals embedded either audiovisual exercise instructions or exercise game contents, whereas ActivOnline, “A web portal” (portal 6), and “A web application” (portal 8) promote physical activity by a monitoring function. The TELEKAT portal and IBMS do not promote exercises directly; however, exercise promotion is included in a part of the TELEKAT intervention. As such, the web portals not only present rehabilitation instructions but also facilitate rehabilitation activities through various functional features. In other words, the underlying concept of web portals is to lower the hurdle and promote rehabilitation to become part of patients’ everyday lives.

Taken together, we propose several key concepts that can be addressed in the development of a telerehabilitation web portal. The advantage of web portals is their ability to unify and provide various information, which can be shared by many stakeholders, including patients’ relatives. By contrast, acquiring data and real-time correspondence often demand an extra system. Regarding the weakness of data acquisition and real-time correspondence, providing patients with a tablet or smartphone with a Bluetooth connection may help solve such problems, as exemplified in some studies [30,40,43,48]. Asynchronous than synchronous rehabilitation is more preferrable for web portals, as it is regarded as less time restrictive and more able to access a rehabilitation program [72-75]. This may suit rehabilitation participants who struggle with disruption to their established routine. The principle of patient-centered care may guide development of the digital platform. Consequently, conducting a participatory design process may be desirable. Theoretical approaches are recommended because theories and models can be used not only during the developing phase but also to analyze outcomes. Moreover, using a theoretical approach enables an intervention to be mapped to existing knowledge. Functional features may be developed upon the association of BCTs or MTs. In addition, other BCTs that were not observed in the 14 portals may be included in future telerehabilitation portals, for example, prompt barrier identification or mindfulness techniques [70,76]. Many different kinds of digital platforms are currently available for telerehabilitation. Reasons for choosing certain platforms, such as web applications, iOS/Android apps, and eHealth device–associated applications, are considered to be knowledge gaps, which should be studied in the future. Differences in platforms can be analyzed from the perspective of data privacy issues. Because web portals often contain highly personal information, choosing a web application involves an important security strategy. Security updates of web applications are done by system providers, and, unlike native apps, little action is required from the user side. This is a great advantage for participants, especially those unfamiliar with digital technologies.

Theoretical approaches were found in more than half of the portals analyzed, and BCTs were often mentioned in those studies. Current knowledge of BCTs, however, may not be sufficient to understand BCTs used for telerehabilitation platforms. For instance, although monitoring/tracking data are associated with BCTs and MTs in several web portal articles [30,31,59], the function is not monitoring behavior itself but rather monitoring physical condition or exercise performance. Developing a new analytical method for theories, BCTs, MTs, and mode of delivery used in telerehabilitation may provide evidence on which to base selection of particular combinations.

Web portals are not only practical tools to collect data or supply exercise instructions. If remote monitoring by health care professionals is the only goal, portals may not be necessary to be developed. Patients with chronic diseases often require lifestyle changes. Through patient-centered functions, web portals deliver various useful support for patients with chronic diseases. Web portals also assist rehabilitation to become part of patients’ daily routines, which is considered a key to guiding successful disease management [44]. Web portals have the potential to facilitate patients in applying a new, healthy lifestyle in accordance with their symptoms and help participants master their own diseases in everyday life.

### Limitations

International consensus about web portals for telerehabilitation is scarce. To effectively extract the characteristics, rather strict inclusion criteria were applied in this study. As mentioned in the “Methods” section, portals developed for purposes other than telerehabilitation were excluded, as were clinician portals, portals targeting other than chronic diseases, web applications designed for a single purpose, and web services with limited time of access. In this manner, we were able to bring out some characteristic features of web portals for telerehabilitation. We believe that our findings can be used in comparative studies of digital platforms of telerehabilitation in future investigations. Another limitation is that some of the projects described in this review are currently ongoing, suggesting that the functions of their portals may be improved in the future. Finally, this study did not include the cost of utilities, or patients’ and health care professionals’ experiences and perspectives when using the portals.

### Conclusions

Telerehabilitation has several advantages, including reducing the need to visit clinics by patients vulnerable to infectious diseases. The global COVID-19 pandemic has increased the need for telerehabilitation. Web portals have the potential to be a core digital component for patient-centered telerehabilitation. The common functional features of 14 web portals studied in this scoping review were monitoring/tracking data provided by 11 portals; communication, 10; exercise instructions, 9; diary/self-report functions, 9; relatives’ participation, 6; goal settings, 5; and education, 5. Although different functional features addressed various purposes, the underlying concept was to facilitate rehabilitation to become a part of patients’ everyday lives. Web portals were able to unify and display multiple types of data and could effectively provide various types of information. Asynchronous correspondence was more favorable than synchronous, real-time interactions. Data acquisition often demanded other digital tools. As much as 8 out of 14 portals employed theoretical approaches to some degree, and various patient-centered functions were observed. As is usual in web applications, security updates were the responsibility of service providers, and thus such platforms may be especially suitable for participants who are unfamiliar with digital technologies. These findings suggested that web portals for telerehabilitation not only provide entrance into rehabilitation programs but also reinforce patient-centered treatment, adherence to rehabilitation, and lifestyle changes over time.

## Appendix

Multimedia Appendix 1 Descriptions of each portal.

## Abbreviations

ActionPACT: the active online physical activity in the cystic fibrosis trial
BCT: behavior change technique
COPD: chronic obstructive pulmonary disease
IBMS: integrated care portal multiple sclerosis
PRISMA-ScR: Preferred Reporting Items for Systematic Reviews and Meta-analysis Extension for Scoping Reviews
PROs: patient-reported outcomes
TELEKAT: telehomecare, chronic patients, and the integrated health care system
TTP: teledialog telerehabilitation program
