# Supplementary material for: Web Portals for Patients With Chronic Diseases: Scoping Review of the Functional Features and Theoretical Frameworks of Telerehabilitation Platforms
Source: J Med Internet Res. 2022 Jan 27;24(1):e27759. doi: 10.2196/27759 (PMC8832270; doi:10.2196/27759)
Supplement: Multimedia Appendix 1 [file jmir_v24i1e27759_app1.docx]

### Appendix 1

### Portal 1. ActiveHeart

ActiveHeart is a web-based portal developed in Denmark for the “Teledialog Telerehabili1tation Program (TTP)” [1]. This portal targets cardiac patients and their relatives, and the language used by the site is Danish [1]. The authors describe the portal as “an interactive portal that functions as a toolbox” [2]. The system was designed through a participatory design process [2]. Feasibility and acceptability were assessed and analyzed [3,4]. The website, which requires individual login to safeguard data privacy, contains information about rehabilitation related issues including medications, smoking, mental well-being, diets, and exercises [1,2]. Information is provided via text, images and videos, including cardiac patients presenting their stories and experiences of rehabilitation [1]. The TTP project also includes a personal computer-based application called “Shared Care Platform” (IBM Corp.), which provides health monitoring functions (blood pressure, pulse, weight and steps) as well as medications, goals, diary, scheduling and plan for rehabilitation [2]. Physical data are collected by MyMedic (Tunstall Healthcare, Aalborg, Denmark) and transferred to a central server. Participants are provided wrist-worn activity tracker (FitBit), a sphygmomanometer and a digital weight scale for health monitoring through Shared Care Platform.

### Portal 2. ActivOnline

ActiveOnline is a web-based physical activity platform developed in Australia as part of the Active Online Physical Activity in Cystic fibrosis Trial (ActionPACT) project [5]. The portal targets people with cystic fibrosis with the aim of increasing opportunity and motivation for physical activities, enabling them to track their physical movement, as well as allowing them to set goals and self-monitor their progress [5]. The same system was later used for COPD patients [6]. Physical activity data is manually input by participants though the web application [7]. The site language is English [8]. The authors describe the portal as a “web-based physical activity platform” [5]. Feasibility and acceptability were previously assessed by interviews and Likert scales [7]. Using a messaging system, participants can communicate with the research team directly. To safeguard data privacy, participants each have a unique username and password, and the system employs 128-bit SSL encryption [7]. Participants’ logs are recorded, with messages sent if login is not observed for three days. Data on activity is determined by participants using their own activity trackers (e.g., FitBit) or mobile phones. Participants are provided a pedometer upon request [5].

### Portal 3. e-Exercise

E-Exercise is a web application developed in The Netherlands. The portal targets patients with hip and/or knee osteoarthritis, with its main functions being to provide physical therapy and education [9]. The site language is Dutch [34]. The authors state that the program “combines the advantages of the digital world with the professional knowledge of the physiotherapist” [10]. Usability was assessed and qualitative data about website usage were used to analyze adherence to the web application [11]. Development of the system was based on “Join2move”, a physical activity intervention project, the usability of which had also been previously assessed [12]. The application contains text- and video-based information [9]. Physical therapists post physical activity exercises every week on individual, password-protected pages [9]. The core method of the exercises is gradually increased activity in a time contingent program [9]. The website includes evaluation of graded activity modules for participants’ minutes of physical activities, rating of pain during activity and difficulty of exercise [13]. The website also includes weekly educational modules that provide new contents about arthritis, exercises and chronic pain [10]. Weekly emails encourage participants to stay the course of their exercise programs [10]. Although, the web application does not have a functional feature to set goals, goals are discussed during face-to-face sessions to generate a tailored exercise program [13]. The 12-week e-Exercise program includes four to five face-to-face sessions to discuss progress with a physiotherapist [10]. A combination of online and face-to-face care (blended care) has been intentionally chosen for successful intervention [9].

### Portal 4. HeartPortal

HeartPortal is a web portal developed as part of the Future Patient-Telerehabilitation of Patients with Heart Failure Project in Denmark [14]. The portal targets patients with heart failure. The site language is Danish [14]. The authors describe the portal as “a digital toolbox that functions as an interactive learning module” [15]. The system was designed through a participatory design process, and its feasibility and acceptability were assessed [14]. Patient education and information on rehabilitation issues, including interviews with patients and their relatives, are presented in text- and video-based forms on an individual, password-protected pages [14]. Patients may communicate directly with their healthcare team via a dialog function [14]. Using the communication platform, patients may set rehabilitation goals and keep an online diary. Participant-reported outcomes (PROs) are evaluated using online questionnaires about sleep patterns, well-being, physical limitations, symptoms, self-efficacy, social interactions and quality of life (QOL) [15]. Each participant is provided a blood pressure monitor, a weight scale, a data transmitter, a step counter, a sleep sensor, and a tablet (iPad). These monitoring devices collect physical data with the results displayed graphically via a data transmitter. All the measured values are stored on a secure database [15]. If allowed by patients, their relatives may log in to the portal. The Heart Portal is being tested in a randomized controlled trial [15].

### Portal 5. Integrated Care Portal Multiple Sclerosis (IBMS)

IBMS is a patient portal developed in Germany based on surveys and workshops with patients and health care professionals (HCPs) [16]. The portal targets patients with multiple sclerosis (MS), and the language used is German [16]. The authors describe the portal as “a digital tool to deliver the care model” [16]. The system was designed through a participatory design process, and its usability is going to be assessed in a future study [16]. The web application includes a dashboard for patients with news and a tracker to record the temporal occurrence of relapses and changes in an individual’s disease course on their password-protected page [16]. The system also contains past and future appointments, medications, current tasks and a symptom diary. PROs are evaluated from information written in the diary. Relatives can log in to the portal if allowed by the patient [16]. The data monitoring / tracking function of IBMS relies on external software. The portal is connected to MS care records and disease-specific software, the multidimensional Multiple Sclerosis Documentation System (MSDS^3D^)[16,17]. The MS care record contains MS-related information on the patients that can be accessed by both patients and HCPs [16]. The software gathers clinical data and laboratory test results, magnetic resonance imaging (MRI) and questionnaires [16]. Through IBMS, patients are able to fill out a patient admission form in preparation for the examination appointment. Information related to the patient-specific pathways and educational materials on the respective pathways is also provided via IBMS [16].

### Portal 6. “A web-portal”

“A web-portal” was developed in The Netherlands to target patients with COPD. Although the language used by the site was not mentioned, participants were required to have Dutch reading skills, which suggests the site language is Dutch [18]. The authors describe the portal as complementing a mobile phone device “activity coach” worn on each participant’s belt. The activity coach collects participants’ activity level using a triaxial accelerometer, providing real-time feedback to the participants. The activity level is displayed on the mobile phone app as well as the web portal. The portal is used to keep a diary and display each individual’s activity data [18]. Using the web portal, participants are expected to become more aware of their own health condition, including the ability to self-treat exacerbations of their disease [18].

### Portal 7. TELEKAT portal

The TELEKAT portal developed in Denmark as part of the Telehomecare project targets patients with chronic conditions and integrates them into the health-care system (TELEKAT) [19-21]. It focuses on patients with COPD [19-21], and the site language is Danish [21]. The authors describe the portal as “technology as a network creator” [20]. The system was designed through a participatory design process [21], and its feasibility and acceptability were analyzed [19,4]. Each participant is provided a digital weight scale, a blood pressure meter, a pulse oximeter, and a spirometer to collect physical data [22]. Health data, including blood pressure, pulse, weight, oxygen level and spirometry, are securely transferred using wireless telehealth monitor, MyMedic/MyMedic plus (Tunstall Healthcare, Aalborg, Denmark) [19]. The data are displayed on the web portal and can be assessed by the patients, their relatives and healthcare professionals across sectors [19]. The portal contains a communication function that allows patients to create a patient-network and to exchange ideas on how to handle their disease. Patients therefore have an opportunity to learn from each other, with this communication function also being an educational function.

### Portal 8. “A web application” (SmartCareCAD project)

“A web application” was developed in The Netherlands as a core component of the intervention, SmartCareCAD project [23]. It targets patients with coronary artery disease [23]. Although the language used by the site was not mentioned, participants were required to have Dutch reading skills, which suggests the site language is Dutch [23]. The authors state this program is “a secured, personalised, patient-centered web application” including goal-setting, communication functions, and data tracking features [23]. The web application adopts two-factor authentication to login; thus, participants are required to have a mobile phone with short message service [23]. Each participant is provided a wrist-worn heart rate monitor (Mio Alpha, Physical Enterprises Inc., Vancouver, British Columbia, Canada) and a hip-worn accelerometer (ActiGraph wGT3x-BT, Actigraph LLC, Pensacola, Florida, USA) [23]. Participants wear a heart rate monitor and accelerometer during exercise sessions and are instructed to wear the accelerometer four days a week during daytime [23]. The heart rate data is sent to the accelerometer by Bluetooth connection and stored locally [23]. Participants are requested to upload the physical data to the web application weekly via USB-connection between the accelerometer and PC [23]. The data are graphically displayed on the web application and can be assessed by the patients, their relatives and healthcare professionals [23]. The web application contains a video conference function that allows participants to have a weekly video consultation with the physical therapist [23]. The web application generates an alert when no data upload, reduced exercise, or reduced physical activity within four weeks [23].

### Portal 9. “A webpage for self-management”

“A webpage for self-management” was developed in Norway to target COPD patients participating in a long-term telerehabilitation intervention, with a 2-year follow-up [24]. Although the language used by the site was not mentioned, participants were required to have Norwegian language ability, which suggests the site language is Norwegian [25]. The authors describe it as a “web-based education/self-management platform”, which includes an individual training program, daily diary, e-form to complete after a training session, and access to historical data [24]. Each participant is provided a treadmill, a pulse oximeter (Nonin GO2 LED), and tablet computer (Apple iPad 2) with holder to attach it to a treadmill [24]. The program completion, Borg scale, oxygen saturation data, and heart rate are transferred by participants completing an e-form on the webpage after each training session [24]. Although the webpage has no communication function, a videoconferencing application (LifeSize, ClearSea) is installed on the provided tablet computer [24]. Participants are requested to perform a training program individually a few times a week on the treadmill and strength training. In addition, each participant receives weekly videoconference sessions with the physiotherapist one-to-one or with other participants [24].

### Portal 10. e-CUIDATE system

E-CUIDATE system was developed in Spain as part of the e-CUIDATE program[26]. The web application targets breast cancer survivors [26,27]. The language used is Spanish [27]. The authors state this program is “an online system that facilitates the development of remote rehabilitation” [26]. The web application consists of a public interface with updated information about breast cancer and a private interface that requires a personal username and password to login [26]. The private section contains exercise instructions, a videoconference system and a messaging function [26]. The web application uses not only exercise based interventions but a telerehabilitation assessment of lymphedema [28]. An additional function for manually entering arm measurement data and instructions on tape measurement technique are described by Galiano-Castillo N, *et al.* [28].

### Portal 11. “a web portal” (Condition Coach program)

“Web portal” was developed in The Netherlands as part of the Condition Coach program [29]. The portal targets patients with COPD [29]. The language is Dutch [29]. Development of the system was based on several studies including “a web portal” (Portal 6) which has been described [18]. The program was designed as blended care, which was implemented within usual care [29]. The application contains exercise instructions, diary functions, a teleconsultation module for consultations between participant and physiotherapist, and displays activity levels [29]. An exercise scheme, including details and frequencies of exercise, is individually prescribed to participants [29]. Participants may use an additional exercise option if they wish [29]. Each participant is provided an accelerometer and smartphone connected by Bluetooth [29]. Participants can see their cumulative measured activity in a graph on the web portal [29]. Motivating messages are sent to the provided smartphone based on participants’ activity level [29].

### Portal 12. “a web platform”

“Web platform” was developed in Spain to target patients with MS [30]. The language is Spanish [30]. The application contains a monitoring module, communication module (forum, blog, videoconference and chat), exercise recording module, and notification center [30]. Authors mentioned use of multimedia technologies like Red5 and Adobe Flex [30], suggesting those open-source software were used to develop the exercise recording module. The system requires an *ad-hoc* desktop application to track the posture of patients during rehabilitation sessions [30]. The system has been designed for a combination of online and face-to-face care (blended care) [30].

### Portal 13. LifeCIT Web site

LifeCIT Web site was developed in UK as part of constraint-induced therapy for people with upper limb disability after stroke (LifeCIT) [31]. Although, neither the language used on the site nor the participants’ language ability were mentioned, use of simple language and minimizing the amount of text were described [31]. The system was designed through a participatory design process using the “person-based approach”[31,32]. Complete instructions and guidance on using the system are included in the web pages [31]. An upper limb function assessment automatically generates activities based on the initial assessment [31]. Guidance on how to obtain maximum benefit from using the system are included among functional features of the web page [31]. The system includes a web-messaging function with a secured password-protected internet connection [31]. Each participant is provided a “C-Mitt”, which was designed to restrict functional hand movement for rehabilitation purpose [31].

### Portal 14. “A website”

“A website” was developed for a long-term two-year telerehabilitation intervention conducted across sites in three countries, Norway, Australia and Denmark [33]. The targeted symptom was COPD. The language used by the site is not mentioned. The authors state this program is “a customised website” for self-management, which includes an individual training program, daily diary, e-form to record a training session, access to historical data, exchange electronic messages, schedule videoconferencing sessions, and assess to individual goal settings and goal attainment [33]. Each participant is provided a treadmill, pulse oximeter (Nonin GO2 LED), and tablet computer (Apple iPad Air) with holder [33]. The program completion, Borg scale, oxygen saturation data, and heart rate are recorded by participants in an e-form after each training session [24]. In addition, a videoconferencing application (LifeSize, ClearSea) is installed on the provided tablet computer [33]. Participants are requested to perform a training program individually three to five times a week on the treadmill, and strength training. In addition, each participant receives weekly videoconference sessions with a physiotherapist in the first eight weeks after enrolment, and at least monthly sessions in the following period [33]. Physiotherapist supervised group exercise sessions can be conducted [33].

**References**

1. Melholt C, Joensson K, Spindler H, Hansen J, Andreasen JJ, Nielsen G, Noergaard A, Tracey A, Thorup C, Kringelholt R, Dinesen BI (2018) Cardiac patients' experiences with a telerehabilitation web portal: Implications for eHealth literacy. Patient Educ Couns 101 (5):854-861. doi:10.1016/j.pec.2017.12.017

2. Dinesen B, Spindler H (2018) The Use of Telerehabilitation Technologies for Cardiac Patients to Improve Rehabilitation Activities and Unify Organizations: Qualitative Study. JMIR Rehabil Assist Technol 5 (2):e10758. doi:10.2196/10758

3. Dinesen B, Nielsen G, Andreasen JJ, Spindler H (2019) Integration of Rehabilitation Activities Into Everyday Life Through Telerehabilitation: Qualitative Study of Cardiac Patients and Their Partners. J Med Internet Res 21 (4):e13281. doi:10.2196/13281

4. Kvale S, Brinkmann S (2009) InterViews: Learning the Craft of Qualitative Research Interviewing. SAGE Publications,

5. Cox NS, Eldridge B, Rawlings S, Dreger J, Corda J, Hauser J, Button BM, Bishop J, Nichols A, Middleton A, Ward N, Dwyer T, Tomlinson OW, Denford S, Barker AR, Williams CA, Kingsley M, O'Halloran P, Holland AE (2019) A web-based intervention to promote physical activity in adolescents and young adults with cystic fibrosis: protocol for a randomized controlled trial. BMC Pulm Med 19 (1):253. doi:10.1186/s12890-019-0942-3

6. Liacos A, Burge AT, Cox NS, Holland AE (2018) Promoting Physical Activity Using the Internet: Is It Feasible and Acceptable for Patients With Chronic Obstructive Pulmonary Disease and Bronchiectasis? J Aging Phys Act 26 (3):372-381. doi:10.1123/japa.2017-0123

7. Cox NS, Alison JA, Button BM, Wilson JW, Holland AE (2015) Feasibility and acceptability of an internet-based program to promote physical activity in adults with cystic fibrosis. Respir Care 60 (3):422-429. doi:10.4187/respcare.03165

8. ActiveOnline Website. http://www.activonline.com.au/ [Accessed May 2021].

9. Bossen D, Kloek C, Snippe HW, Dekker J, de Bakker D, Veenhof C (2016) A Blended Intervention for Patients With Knee and Hip Osteoarthritis in the Physical Therapy Practice: Development and a Pilot Study. JMIR Res Protoc 5 (1):e32. doi:10.2196/resprot.5049

10. e-Exercise introduction video. https://www.youtube.com/watch?v=4l9GoQWWy58. [Accessed May 2021].

11. Kloek CJJ, Bossen D, Spreeuwenberg PM, Dekker J, de Bakker DH, Veenhof C (2018) Effectiveness of a Blended Physical Therapist Intervention in People With Hip Osteoarthritis, Knee Osteoarthritis, or Both: A Cluster-Randomized Controlled Trial. Phys Ther 98 (7):560-570. doi:10.1093/ptj/pzy045

12. Bossen D, Veenhof C, Dekker J, de Bakker D (2013) The usability and preliminary effectiveness of a web-based physical activity intervention in patients with knee and/or hip osteoarthritis. BMC Med Inform Decis Mak 13:61. doi:10.1186/1472-6947-13-61

13. de Vries HJ, Kloek CJJ, de Bakker DH, Dekker J, Bossen D, Veenhof C (2017) Determinants of Adherence to the Online Component of a Blended Intervention for Patients with Hip and/or Knee Osteoarthritis: A Mixed Methods Study Embedded in the e-Exercise Trial. Telemed J E Health 23 (12):1002-1010. doi:10.1089/tmj.2016.0264

14. Joensson K, Melholt C, Hansen J, Leth S, Spindler H, Olsen MV, Dinesen B (2019) Listening to the patients: using participatory design in the development of a cardiac telerehabilitation web portal. Mhealth 5:33. doi:10.21037/mhealth.2019.08.06

15. Dinesen B, Dittmann L, Gade JD, Jørgensen CK, Hollingdal M, Leth S, Melholt C, Spindler H, Refsgaard J (2019) "Future Patient" Telerehabilitation for Patients With Heart Failure: Protocol for a Randomized Controlled Trial. JMIR Res Protoc 8 (9):e14517. doi:10.2196/14517

16. Voigt I, Benedict M, Susky M, Scheplitz T, Frankowitz S, Kern R, Müller O, Schlieter H, Ziemssen T (2020) A Digital Patient Portal for Patients With Multiple Sclerosis. Front Neurol 11:400. doi:10.3389/fneur.2020.00400

17. Ziemssen T, Kempcke R, Eulitz M, Großmann L, Suhrbier A, Thomas K, Schultheiss T (2013) Multiple sclerosis documentation system (MSDS): moving from documentation to management of MS patients. J Neural Transm (Vienna) 120 Suppl 1:S61-66. doi:10.1007/s00702-013-1041-x

18. Tabak M, Vollenbroek-Hutten MM, van der Valk PD, van der Palen J, Hermens HJ (2014) A telerehabilitation intervention for patients with Chronic Obstructive Pulmonary Disease: a randomized controlled pilot trial. Clin Rehabil 28 (6):582-591. doi:10.1177/0269215513512495

19. Dinesen B, Huniche L, Toft E (2013) Attitudes of COPD patients towards tele-rehabilitation: a cross-sector case study. Int J Environ Res Public Health 10 (11):6184-6198. doi:10.3390/ijerph10116184

20. Dinesen B, Andersen SK, Hejlesen O, Toft E (2011) Interaction between COPD patients and healthcare professionals in a cross-sector tele-rehabilitation programme. Stud Health Technol Inform 169:28-32

21. Dinesen B, Seeman J, Gustafsson J (2011) Development of a program for tele-rehabilitation of COPD patients across sectors: co-innovation in a network. Int J Integr Care 11:e012. doi:10.5334/ijic.582

22. Jensen MH, Cichosz SL, Dinesen B, Hejlesen OK (2012) Moving prediction of exacerbation in chronic obstructive pulmonary disease for patients in telecare. J Telemed Telecare 18 (2):99-103. doi:10.1258/jtt.2011.110607

23. Brouwers RW, Kraal JJ, Traa SC, Spee RF, Oostveen LM, Kemps HM (2017) Effects of cardiac telerehabilitation in patients with coronary artery disease using a personalised patient-centred web application: protocol for the SmartCare-CAD randomised controlled trial. BMC Cardiovasc Disord 17 (1):46. doi:10.1186/s12872-017-0477-6

24. Zanaboni P, Lien LA, Hjalmarsen A, Wootton R (2013) Long-term telerehabilitation of COPD patients in their homes: interim results from a pilot study in Northern Norway. J Telemed Telecare 19 (7):425-429. doi:10.1177/1357633x13506514

25. Hoaas H, Andreassen HK, Lien LA, Hjalmarsen A, Zanaboni P (2016) Adherence and factors affecting satisfaction in long-term telerehabilitation for patients with chronic obstructive pulmonary disease: a mixed methods study. BMC Med Inform Decis Mak 16:26. doi:10.1186/s12911-016-0264-9

26. Galiano-Castillo N, Cantarero-Villanueva I, Fernández-Lao C, Ariza-García A, Díaz-Rodríguez L, Del-Moral-Ávila R, Arroyo-Morales M (2016) Telehealth system: A randomized controlled trial evaluating the impact of an internet-based exercise intervention on quality of life, pain, muscle strength, and fatigue in breast cancer survivors. Cancer 122 (20):3166-3174. doi:10.1002/cncr.30172

27. Galiano-Castillo N, Arroyo-Morales M, Lozano-Lozano M, Fernández-Lao C, Martín-Martín L, Del-Moral-Ávila R, Cantarero-Villanueva I (2017) Effect of an Internet-based telehealth system on functional capacity and cognition in breast cancer survivors: a secondary analysis of a randomized controlled trial. Support Care Cancer 25 (11):3551-3559. doi:10.1007/s00520-017-3782-9

28. Galiano-Castillo N, Ariza-García A, Cantarero-Villanueva I, Fernández-Lao C, Sánchez-Salado C, Arroyo-Morales M (2014) Agreement between telerehabilitation involving caregivers and face-to-face clinical assessment of lymphedema in breast cancer survivors. Support Care Cancer 22 (1):253-258. doi:10.1007/s00520-013-1971-8

29. Tabak M, Brusse-Keizer M, van der Valk P, Hermens H, Vollenbroek-Hutten M (2014) A telehealth program for self-management of COPD exacerbations and promotion of an active lifestyle: a pilot randomized controlled trial. Int J Chron Obstruct Pulmon Dis 9:935-944. doi:10.2147/COPD.S60179

30. Eguiluz-Perez G, Garcia-Zapirain B (2014) Comprehensive verticality analysis and web-based rehabilitation system for people with multiple sclerosis with supervised medical monitoring. Biomed Mater Eng 24 (6):3493-3502. doi:10.3233/BME-141175

31. Burridge JH, Lee ACW, Turk R, Stokes M, Whitall J, Vaidyanathan R, Clatworthy P, Hughes AM, Meagher C, Franco E, Yardley L (2017) Telehealth, Wearable Sensors, and the Internet: Will They Improve Stroke Outcomes Through Increased Intensity of Therapy, Motivation, and Adherence to Rehabilitation Programs? J Neurol Phys Ther 41 Suppl 3:S32-S38. doi:10.1097/NPT.0000000000000183

32. Yardley L, Morrison L, Bradbury K, Muller I (2015) The person-based approach to intervention development: application to digital health-related behavior change interventions. J Med Internet Res 17 (1):e30. doi:10.2196/jmir.4055

33. Zanaboni P, Dinesen B, Hjalmarsen A, Hoaas H, Holland AE, Oliveira CC, Wootton R (2016) Long-term integrated telerehabilitation of COPD Patients: a multicentre randomised controlled trial (iTrain). BMC Pulm Med 16 (1):126. doi:10.1186/s12890-016-0288-z
